# Using Simulation Models to Evaluate Ape Nest Survey Techniques

**DOI:** 10.1371/journal.pone.0010754

**Published:** 2010-05-21

**Authors:** Ryan H. Boyko, Andrew J. Marshall

**Affiliations:** Department of Anthropology and Graduate Group in Ecology, University of California Davis, Davis, California, United States of America; University of Zurich, Switzerland

## Abstract

**Background:**

Conservationists frequently use nest count surveys to estimate great ape population densities, yet the accuracy and precision of the resulting estimates are difficult to assess.

**Methodology/Principal Findings:**

We used mathematical simulations to model nest building behavior in an orangutan population to compare the quality of the population size estimates produced by two of the commonly used nest count methods, the ‘marked recount method’ and the ‘matrix method.’ We found that when observers missed even small proportions of nests in the first survey, the marked recount method produced large overestimates of the population size. Regardless of observer reliability, the matrix method produced substantial overestimates of the population size when surveying effort was low. With high observer reliability, both methods required surveying approximately 0.26% of the study area (0.26 km^2^ out of 100 km^2^ in this simulation) to achieve an accurate estimate of population size; at or above this sampling effort both methods produced estimates within 33% of the true population size 50% of the time. Both methods showed diminishing returns at survey efforts above 0.26% of the study area. The use of published nest decay estimates derived from other sites resulted in widely varying population size estimates that spanned nearly an entire order of magnitude. The marked recount method proved much better at detecting population declines, detecting 5% declines nearly 80% of the time even in the first year of decline.

**Conclusions/Significance:**

These results highlight the fact that neither nest surveying method produces highly reliable population size estimates with any reasonable surveying effort, though either method could be used to obtain a gross population size estimate in an area. Conservation managers should determine if the quality of these estimates are worth the money and effort required to produce them, and should generally limit surveying effort to 0.26% of the study area, unless specific management goals require more intensive sampling. Using site- and time- specific nest decay rates (or the marked recount method) are essential for accurate population size estimation. Marked recount survey methods with sufficient sampling effort hold promise for detecting population declines.

## Introduction

Nest counts have been used extensively to estimate great ape population densities [1]–[3]. In orangutans, these density estimates have been employed to assess population size, monitor population status, characterize movements among habitats, evaluate conservation tactics, and identify priority populations for conservation [4]–[10]. Rapid nest count methods are particularly important for attaining some conservation goals [11], but the accuracy of these methods has recently been called into question [12]–[14]. Of particular concern is our inability to accurately estimate the amount of time a nest is visible, or the nest decay time. Nest decay times vary substantially over time and among sites and can introduce substantial errors in estimates of orangutan population density [12], [13]. The two methods that are commonly used to estimate orangutan population density address the problem of assessing nest decay time in different ways.

One method of estimating nest density, developed by Hashimoto [1] for chimpanzees, involves counting nests along the same transect at two different times and using only the new nests built after the initial survey to estimate population density. This ‘marked recount’ method (after [13]) precludes the necessity of measuring nest decay time at all, provided that the period between surveys is less than the minimum nest decay time, i.e. provided no nests made after the first survey disappeared prior to the second survey. If nests were to be built and completely decay between surveys, orangutan density would be underestimated [13]. This method assumes that all nests present at the time of the initial count are detected. If nests are missed on the first count and detected on subsequent counts, orangutan density would be overestimated. One shortcoming of this method is that all nests that are spotted on the original survey are excluded from the analysis, resulting in a relatively small sample size for a given survey effort. In addition, the method is sensitive to short-term movements of orangutans as it counts only nests built in a specific area over a relatively short period [5], [13].

An alternative method for estimating orangutan density uses a Markov chain analysis to estimate nest decay time by estimating transition rates between each state in a matrix [2], [15]. This ‘matrix method’ permits use of data from all nests discovered and has been suggested as a valuable tool for rapid nest surveys, since data from only two surveys can be used to estimate decay parameters [5], [11]. However, this method requires the estimation of several parameters and its reliability depends on observing the complete decomposition of many nests, which is generally impractical over the course of a short survey [2], [13]. Therefore, the point estimate of nest decay time that results from this method is prone to substantial error [13]. An alternative tactic that is frequently used for quickly estimating orangutan population size involves using published nest decay rates from other sites instead of using the matrix method to calculate them for the particular sampled site [14]. This method introduces a host of new potential biases and determining which published decay rate to use is problematic at best [14].

As the results of orangutan nest surveys are widely used to allocate conservation effort and assess management techniques, comparisons of the accuracy and precision of different nest survey methods are urgently needed. While alternative nest count methods have been compared in the field [1], limited sample sizes, small numbers of independent replicates, and an inability to examine variation in survey parameters have hampered investigators' abilities to identify the relative strengths and weaknesses of alternative survey methods. Here we use simulation modeling to examine the accuracy and precision of orangutan population estimates obtained using marked recount and matrix methods, and consider the influence of sampling frequency and intensity on these estimates. We also consider how using published decay rates from other sites instead of assessing decay rates at the survey site affect population size estimates.

## Methods

### Simulation

RHB coded the simulation in the C++ programming language, compiled using wxDev-Cpp (Version 2.6.2; http://bloodshed.net/dev/devcpp.html). We ran the simulations on a PC running Windows Vista SP1.

### Simulation environment

The simulation environment consisted of a 5000×5000 grid of squares, with each square representing a 2 m×2 m area. At the start of a simulation run, we randomly populated each square with a tree of a certain type. We used four different types of trees, each with an equal (¼) probability of occupying a given square. We also modeled an altitudinal gradient where elevation gradually sloped across the x-axis (see below).

We set the mean and variance in nest decay rate in each of five nest decay classes (A, B, C, D and E; modeled after [13]) at the start of each simulation (see Table 1). From these distributions, we randomly drew the mean nest decay rate for each of the four tree types at the start of each simulation. (thus it varied between simulation runs) The variance in nest decay rate for each tree type was set at half the mean type-specific decay rate. Each nest was given a decay rate for each category (A–E) drawn from the tree type-specific normal distribution of decay times.

**Table 1:** Parameter values used in the simulation.

Parameter	Value	Source
A->B Decay Rate	5 +/− 2.5 Days	pers. obs.
B->C Decay Rate	20 +/− 10 Days	pers. obs.
C->D Decay Rate	40 +/− 20 Days	pers. obs.
D->E Decay Rate	80 +/− 40 Days	pers. obs.
E->Disappear Decay Rate	120 +/− 60 Days	pers. obs.
Overall Decay Rate	265 Days (Range: 53.5–760)	[13]
Orangutan Day Range	700 +/− 200 m	[16]
Day nests per day	0.2	[16]
Day nest multiplier	1.667	[17], pers. obs.
Daily seasonal decay factor change	0 +/− 0.05	[13]
Seasonal decay factor	0.625–1.6	[13]
Census interval	30 days	[13]
Census strip width	20 m	[13]
P(detect stage A nest)	0.9	[7]
P(detect stage B nest)	0.95	[7]
P(detect stage C nest)	0.95	[7]
P(detect stage D nest)	0.85	[7]
P(detect stage E nest)	0.8	[7]

The parameters used in running the nest building, decaying and censusing simulation.

As altitude is known to affect nest decay rates [7], an altitudinal gradient was modeled as a multiplier to the tree type-specific decay rate such that moving along the x-axis the multiplier linearly increased according to the equation:
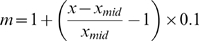
(1)where m is the altitudinal multiplier and x_mid_ is the x coordinate value in the middle of the environment.

The values we used correspond to about a 370 meter change in altitude over the simulation area [7]. Thus, after each nest was given a tree species-specific decay rate, that rate was multiplied by the altitudinal multiplier.

### Orangutans

At the start of the simulation, 200 orangutans were randomly distributed in the simulated habitat, resulting in a population density of two orangutans/km^2^, consistent with typical population densities for wild Bornean orangutans [10], [13]. All orangutans in our simulation built nests, therefore this population should be thought of as containing only adults. At each time step, corresponding to a day, each orangutan moved a set distance (i.e., number of squares) that was drawn randomly from a normal distribution with mean 350 squares (700 meters) and variance 100 squares (see Table 1; [16]). Each move was made randomly in one of the eight possible directions (including diagonal moves), except where the orangutan was at an edge of the simulation environment. In this case, the boundary was reflecting and so the move was forced to be in a direction away from the edge. After each move, orangutans built a day nest with a low probability, such that orangutans built an average of 0.2 day nests per day (Table 1, [16]). These nests were treated exactly the same as night nests except they decayed more quickly; each A–E category decay rate was multiplied by a ‘day nest factor’ (Table 1). After moving the full distance for the day, orangutans built a night nest (as noted above, in this simulation, all orangutans build night nests; dependent infants and juveniles were not modeled). At the time of nest construction, the nest decay parameters for that particular nest were set (see simulation environment section). Any nests created on a square with an existing nest were modeled as ‘reused’ nests such that the old nest was completely replaced with the new nest (this only occurred to about 1% of nests; data not shown). Exact rates of night nest reuse vary among sites (e.g., 4% in Sebangau-13% at Kinabatangan [18]), but in the vast majority of cases, nests are rebuilt when they are reused (e.g., of the 10.9% of total nests that were reused or rebuilt at Tuanan, 99% of these were rebuilt nests and only 1% were simply reused). This suggests that our modeling of nest reuse as replacement is highly concordant with observations of nest building behavior in wild orangutans.

### Nest decay

At each time step, each nest decayed from one state to the next with a certain probability. This probability was based on the predetermined nest decay parameters for that particular nest multiplied by a ‘seasonal decay’ factor. This seasonal decay factor modeled temporally autocorrelated differences in decay rate based on environmental factors (e.g., rainfall; [13]). Each ‘day’, a random number was drawn from a normal distribution with mean 0 and variance 0.05 (see Table 1). This number was then added or subtracted from the last day's seasonal decay factor (or 1.0 on the first day), creating a series of temporally correlated numbers that were bounded between 0.625 and 1.6. Thus, the seasonal decay factor could result in changes in actual decay probabilities of up to 2.56 times over time, consistent with the differences reported in [13].

### Nest surveys – general methods

Starting at day 1000, nest surveys occurred at regular, thirty day intervals. Each survey involved a certain number of survey transects of a given length and a width of ten squares (twenty meters; Table 1; [13]). For the first surveys in a given simulation, we randomly drew a starting point for each transect, with the provision that the entire transect must fit within the simulation environment. We conducted subsequent surveys in the same simulation run along the same transects. We recorded nests along the entire length and width of the transect. For ‘incomplete visibility’ simulations, nests that were undetected on the initial survey were ‘discovered’ on subsequent surveys with a certain probability dependent on their decay stage (Table 1; [7]); all nests detected on a survey were recorded on all subsequent surveys, simulating the tagging of the original nest. Johnson et al. [7] found that survey teams missed a significant proportion of nests on a first survey (16.2% to 34.7% across thirteen sites), so these values (5% to 20% of nests depending on decay stage) conservatively estimated how many nests may be missed by survey crews (though survey crew training and experience likely play a part in determining the proportion of nests missed). These estimates were broadly consistent with the detection probabilities found by [8]. In ‘complete visibility’ simulations, all nests were seen with probability 0.99, except nests that had been detected on previous surveys, which were always found. The complete visibility simulations modeled the use of very well-trained and careful observers or resurvey methods to catch missed nests.

### Nest surveys – Marked recount method

For the marked recount method, we made a minimum of two surveys along a given transect. At the second and any subsequent surveys, we counted the number of new nests discovered (including reused nests that were at an earlier stage of decay). Based on these nest counts, we calculated nest density according to the following equation:
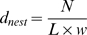
(2)where N  =  the number of nests observed, L  =  the transect length and w  =  the transect width. We then used the equation from [1], reproduced in modified form below as equation (3), to calculate the density of orangutans (d_orang_) in the simulation area:
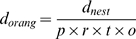
(3)where d_nest_ = density of newly built nests, p = proportion of orangutans that build nests, r = rate of nest building (number of nests built per orangutan per day), t = number of days between surveys, and o = observer skill (proportion of nests seen).

For these calculations, we used p = 1, r = 1.2 and o = the average of the proportion of nests in decay stage A and decay stage B that were detected (as most new nests were in those stages). Thus, these parameters introduced no biases into the results, though in real systems inaccurate estimates of them could lead to biases.

We then calculated the number of orangutans in the simulation environment by multiplying the density by the simulation area (100,000,000 m^2^, or 100 km^2^).

### Nest surveys – Matrix method

The matrix method also required that at least two surveys be made, but with the additional requirement that the amount of time between surveys must be sufficiently large to permit many nests to completely decay [2]. For this method, we recorded each nest, whether new or old, on each transect, along with its decay stage. From these data, we created a transition matrix, **Q**, and calculated the fundamental matrix of **Q**, **N**, using the formula
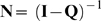
(4)Summing across a row in the fundamental matrix gave the total amount of time a nest was expected to be visible from a certain starting state. Thus, taking the time from stage A yielded the total expected time a nest was visible, i.e., the nest decay time [2]. We multiplied these nest decay times by a ‘correction factor’ to account for the decreased probability that short-lived nests would be seen. We used the correction factor of 0.89, after [7] and [13].

As in the marked recount method, the nest density was calculated from the nest counts using equation (2). However, unlike in the marked recount method, all nests were included, not just new nests. From the nest density, orangutan density was calculated using equation (3), with two slight modifications. First, in this case t stood for the estimated decay time, not the period between surveys. Second, o was calculated as the average observation probability across all nest decay stages, not just stages A and B. Finally, the total number of orangutans was calculated from the orangutan density in the same manner as in the marked recount method.

### Study duration and sampling effort

To examine systematic error in the median estimate for each method under conditions that simulated imperfect observation, we ran several simulations varying the number of surveys from two to ten and the sampling effort from six km (six 1 km transects) to fifty km (ten 5 km transects) under ‘incomplete visibility.’

To determine how the accuracy of each sampling method was affected by differences in study duration and sampling effort, we independently varied the two under ‘complete visibility.’ We considered study durations between two and ten surveys (or, equivalently, two and ten months) and sampling effort between one and ten transects (each 500 m long). We simulated each combination 100 times to produce a distribution of estimates for that combination.

### Use of published decay rate estimates

In order to assess the effect of using published decay rate estimates from other sites instead of directly determining the decay rate at the survey site, we calculated orangutan population size using the survey data from the matrix method with published average nest decay times instead of the decay time calculated in our simulation.

### Population decline

To test the ability of the two nest count methods to detect population decline, we simulated the removal of 5% of the population every year by removing 5% of the remaining population on the first day of each year. Starting on the 1000^th^ day, we conducted surveys monthly for seven years. We used the first four surveys to calculate a baseline population estimate and grouped subsequent surveys into sets of four to re-estimate the population size every four months. We coded each population estimate as a binary variable: it was either greater than or less than the baseline population estimate. As both methods provide unbiased estimates of the mean, we would expect that on average at each observation period the population would be correctly estimated. Therefore, instead of calculating mean population estimates, we use a binary variable to determine how often these methods would correctly raise a flag with conservation managers that the population may be declining and that more detailed study would be in order. We conducted this simulation 100 times and calculated the proportion of population estimates that detected a decline after each four month period and compared it to the null expectation (i.e., a detecting a decline 50% of the time). We averaged across population estimates to calculate the proportion of population estimates that detected a decline during each year of the simulation.

We conducted two simulations, one sampling six 500 meter transects (0.06% of the study area) to simulate low-intensity sampling and one sampling thirteen 1 kilometer transects (0.26% of the study area) to simulate sampling effort that followed our sampling guidelines established by our constant population size models.

### Statistics

To determine whether methods were more likely than chance to detect a decline, we used two-tailed binomial tests since, in real life, surveyors would not know a priori whether a given population was increasing or declining.

## Results

### Population estimates with incomplete visibility

**Figure 1:**
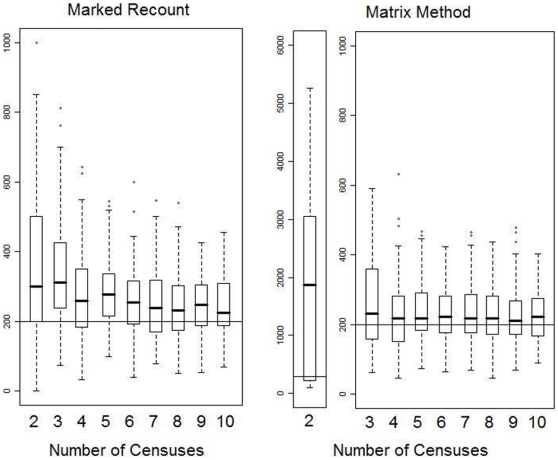
Effect of varying the number of surveys on the accuracy and precision of population estimates. True population size was 200 individuals, indicated by the horizontal line. Data were obtained using six 1 km transects and ‘incomplete visibility’, though the results for different numbers and lengths of transect did not qualitatively differ from these results in terms of medians and general characteristics (data not shown). The median estimates found by the matrix method overestimated the actual number of orangutans by about 5–10%, possibly because temporal changes in decay rate based on differences in decay rate in different stages and in different seasons overshadowed nest-specific decay rate differences. This would mean that, contrary to the expectation that long-lasting nests would be oversampled (as short-lived nests would more frequently be missed), there was not actually a tendency to find nests that would be longer-lasting in the future and thus no correction factor was necessary. The results for the matrix method for two surveys vary widely because so few (or no) nests completely decayed during the study. Note that scales on the y-axis vary among the panels.

The clearest effect under conditions of incomplete visibility was that the marked recount method greatly overestimated population size when only a few surveys were conducted (Fig. 1). Because the standing crop of nests at the first survey was large compared to the number of nests created between surveys, missing about 10–15% of the standing nests at the first survey but finding many of them on subsequent surveys resulted in overestimation of the population size by about 50–75% when only two surveys were conducted. As the number of surveys increased, these nests were integrated into ever-larger numbers of new nests and they consequently had a diminishing impact on the median estimate.

These simulations also highlight the imprecision of using the matrix method when few nests have disappeared (i.e., when few have decayed past the final stage). With only two surveys, few nests had decayed and thus population estimates varied widely and the median estimate was a great overestimate (Fig. 1). This was because in simulations where no nest decayed completely, a population size estimate was impossible as it would have required division by zero. Thus, only those simulations in which at least one nest that had completely decayed were included, seriously underestimating nest decay time and thus overestimating population size.

### Population estimates with complete visibility

**Figure 2:**
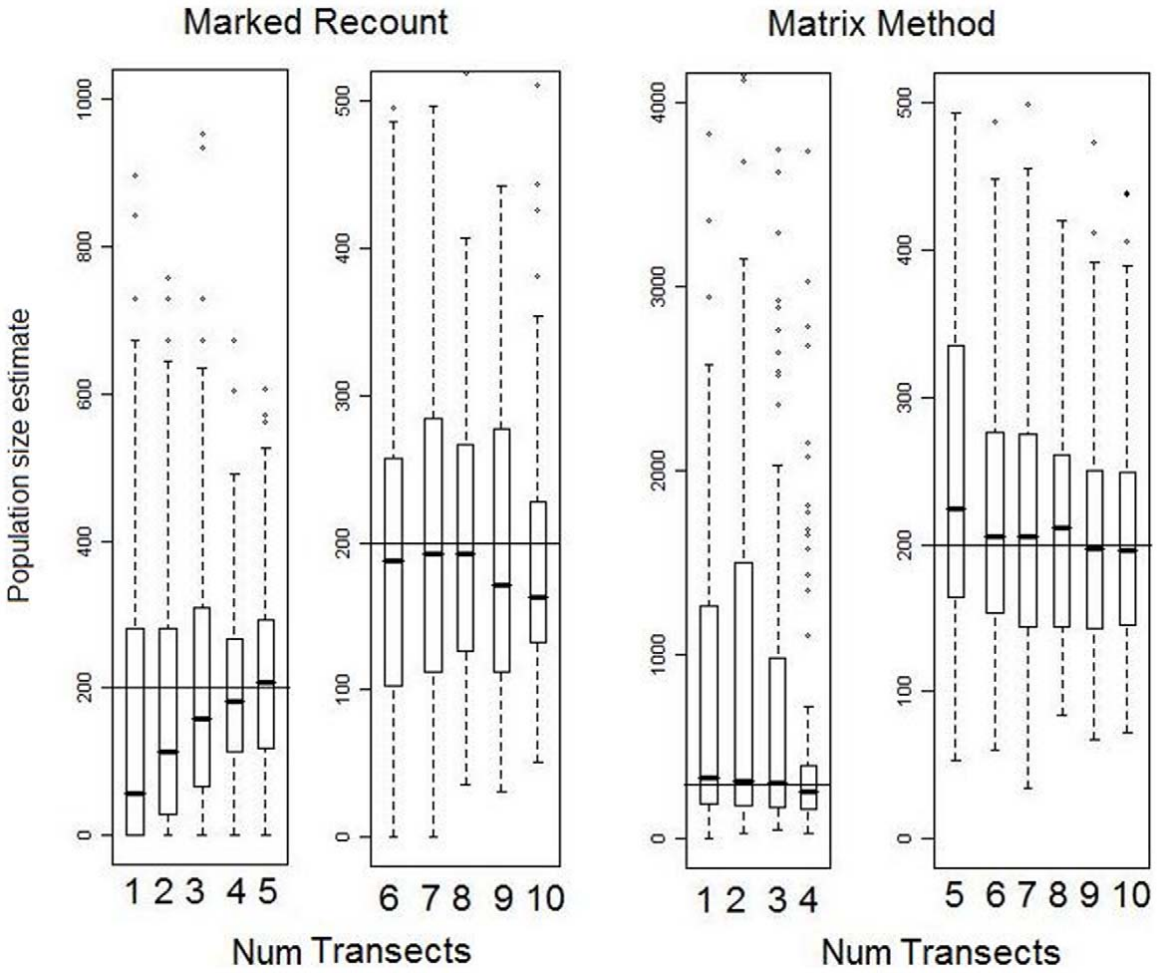
Effect of varying transect number on accuracy and precision of orangutan population estimates. All data were collected with 6 surveys and 500 meter transects. The marked recount method was much more precise with low numbers of transects, though this difference vanished at higher numbers of transects. The matrix method was more accurate however, with the median values more consistently close to the true population size of 200 individuals. These results are similar to those found with 2, 4, 8 and 10 surveys. A few outliers (<1% of the total data points) from both methods not shown. Note that scales on the y-axis vary among the panels.

**Figure 3:**
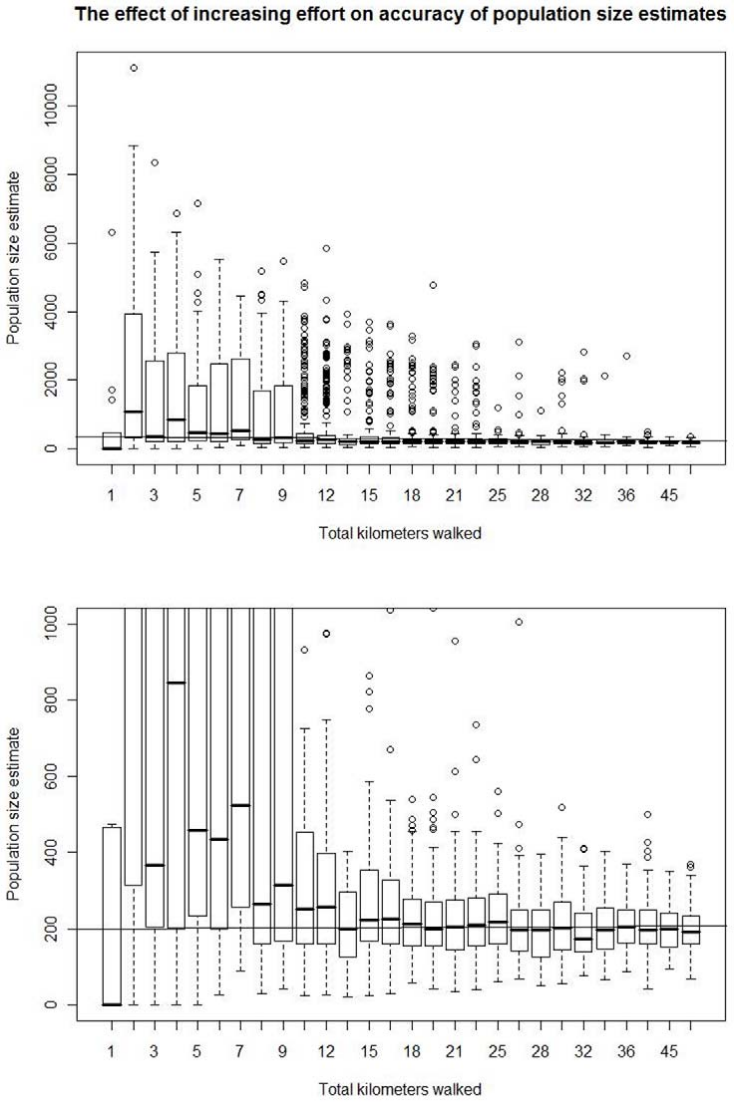
The effect of increasing survey effort on population estimates obtained using the matrix method. The total kilometers walked is the sum of the total transect length times the number of surveys. Increasing the effort above thirteen kilometers (or 0.26 km^2^) provided little increase in performance under the model parameters used. The two panels present the same results, but using different scales on the y-axis.

**Figure 4:**
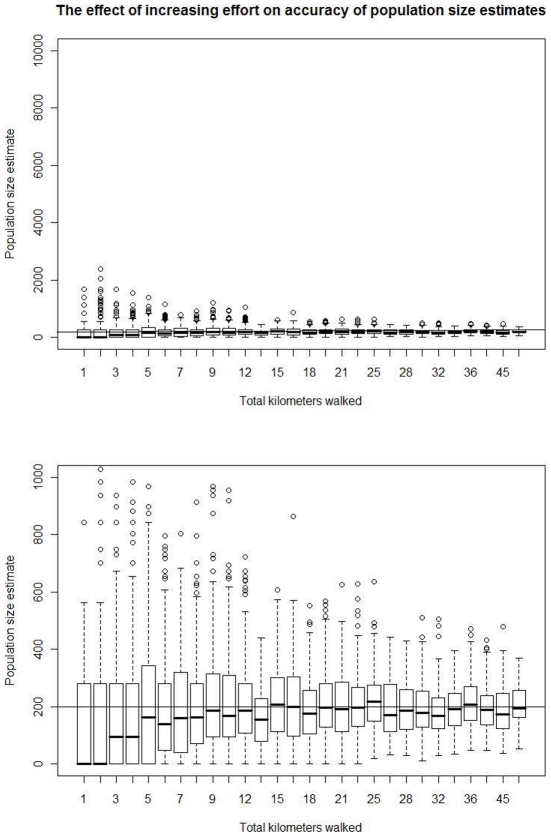
The effect of increasing effort on population estimates obtained using the marked recount method. The total kilometers walked is the sum of the total transect length times the number of surveys. Increasing the effort above thirteen kilometers (or 0.26 km^2^) provided little increase in performance under the model parameters used. The two panels present the same results, but using different scales on the y-axis.

The accuracy and precision of population estimates using both methods increased with increasing numbers of surveys and transects, though this increase was not linear (Figs. 2, 3, 4). As noted above, because of mathematical limitations, the matrix method had large variability and usually overestimated orangutan population size when the number of surveys or transects was low (low surveying effort). Alternatively, the marked recount method tended to underestimate population size with low surveying effort, although there was much variability in these estimates. The reason for this underestimate is less clear, though it may be that the mean is nearly correct while the median is an underestimate because of the high probability of finding no or very few nests due to random clumping since orangutans move a limited distance in a single day.

While the methods differed in their qualitative behavior at low surveying effort, both methods converged on an unbiased estimate of population size and an interquartile range approximately two-thirds the population size at an effort of thirteen survey kilometers (0.26 km^2^, or 0.26% of the total area) spread over two or more surveys (Figs. 3–4). This result did not seem to depend on whether many transects of short distance or few transects of long distance were used (data not shown).

Above a total surveying effort of 0.26 km^2^ (thirteen kilometers in this paper, given the transect width used), more surveying effort provided diminishing returns (Figs. 3–4). For example, in Figures 3 and 4, a total surveying effort of 45 survey kilometers resulted in an interquartile range of about two-thirds the population size using the marked recount method and over one-half of the population size using the matrix method. In general, the matrix method provided moderate improvement in precision with increased surveying effort above thirteen kilometers while the marked recount method improved little if at all.

### Population estimates using decay time estimates from other studies

As published average orangutan nest decay rates at different sites vary from 72 to 424 days [13], we determined how using previously published decay rate data within this range instead of calculating decay rate directly would affect the orangutan population size estimate found using the matrix method. Within this range, we obtained average estimates ranging from 125 to 736 (recall the true population size was 200 individuals) at high sampling effort (i.e. at efforts that produce fairly accurate and unbiased estimates using nest decay data collected during the simulation).

### Detecting population declines

**Figure 5:**
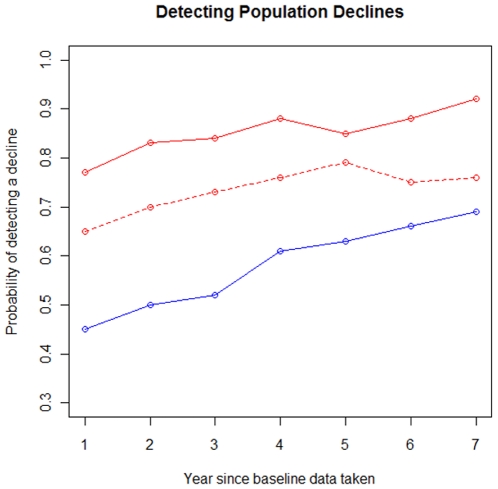
The probability of detecting a population decline increases approximately linearly with the amount of decline. Each year, 5% of the simulated population was removed (“killed”). surveys were conducted monthly; every four months a new population estimate was made based on the previous four surveys. The red lines indicate the proportion of time the marked recount method detected a decline (dashed line used low sampling effort of 0.06% of the study area; solid line used recommended sampling effort of 0.26% of the study area). The blue line indicates the proportion of time the matrix method detected a decline (solid line used the recommended sampling effort of 0.26% of the study area; there is no dashed line as the low sampling effort resulted in a large number of cases without a baseline estimate as no nests decayed completely during the baseline survey). The probability of detecting a decline is the proportion of 100 simulation runs in which a population estimate at that time period was lower than the initial population estimate. For graphical clarity, each data point is based on the average of three consecutive population estimate time periods; hence, it uses one year's worth of data.

The marked recount method had a much higher probability of detecting a population decline of 5% per year, particularly when sampling 0.26% of the study area (Fig. 5). Because of the large number of cases (>10%) in which no nest completely decayed during the baseline sampling for the matrix method when using low sampling intensity, estimates of its accuracy are not included. At low sampling intensity, the matrix method was only somewhat sensitive to detecting population declines. Even after seven years of 5% annual population decline, it was only about 50% more likely than chance to detect a population decline (binomial p<0.01). Even with intensive sampling, the matrix method was not particularly sensitive to population declines, detecting them about 40% more likely than chance after seven years of 5% annual population declines (binomial p<0.01). The large variation in population estimates due to many forms of stochasticity (e.g. seasonal differences in decay rate, sampling bias and inter-nest variation in decay rate) appear to make it difficult to detect even large changes in population size. Averaging over a greater number of surveys, particularly in creating the baseline population estimate, may partially ameliorate this.

However, at the recommended sampling effort level of 0.26% of the study area, the marked recount method does perform fairly well at detecting population declines (Fig. 5). Even after one year, the method detected declines 77% of the time; after seven years this increased to 92% of the time (binomial p<0.01).

## Discussion

We used simulated data of over 700 million nests to determine the accuracy and precision of two population estimation methods, the ‘marked recount’ method and the ‘matrix’ method. At sampling efforts around 0.26% of the study area (thirteen kilometers of transect in this study, or 0.26 km^2^), both the marked recount method and the matrix method produced results of reasonable accuracy and precision. At sampling efforts below this level, neither method produced accurate or precise results: the marked recount method tended to produce underestimates while the matrix method tended to produce overestimates, and the range of estimates returned were very wide for both methods. Increasing the sampling effort above thirteen kilometers yielded little gain in accuracy or precision, though doing so incrementally increased the precision of the matrix method. It is unlikely that incremental increase in precision would be worth additional effort and cost, though that decision would depend on the specific goals of the population survey and the level of funding available. Note that the particular surveying effort required for the marked recount method does not depend on decay time (so long as it is much longer than the inter-survey period) while the required surveying effort for accurate results using the matrix method would increase with longer decay times (as its accuracy depends on including many nests that completely decay). While this recommendation works at most reasonable orangutan densities, at very low orangutan densities, surveying a somewhat larger area may be necessary to obtain a large enough sample size for accurate estimation. This study also confirms the contention that determining the decay rate at a specific site is crucial for accurately using the matrix method to estimate orangutan population size there [14]; using other published average decay rates results in estimates as low as 63% and as high as 368% of the actual orangutan population size.

These results suggest that it does not matter whether one conducts many short surveys or few long surveys. This result should be interpreted with caution, however, for two reasons. First, the orangutans in this simulation moved randomly, which wild orangutans are unlikely to do. Second, tree types were randomly distributed in this environment, although actual habitats in the wild probably vary in a spatially autocorrelated manner. Given the variation in nest decay rates and visibility in different forest types [7], [14], these facts suggest that for a given survey effort (i.e., distance), walking several transects of intermediate length would produce a more accurate estimate than walking a single, longer transect when estimating the size of real populations. The transects used in this study were randomly located and conducted along straight lines, though due to the random movement of orangutans and random distribution of tree types, this was not necessary for accurate results in this simulation. In field situations, however, it is important to use properly randomized or stratified sampling strategies that result in the inclusion of representative amounts of each forest type and account for other important differences across the habitat, such as altitudinal gradients [7]. If deviations from straight-line sampling are influenced by factors that change orangutan behavior, nest decay rates or visibility, such as differences in vegetation type or density, then maintaining straight-line transects will also be important.

These results depend on seeing all or nearly all of the nests. If a non-negligible proportion of nests are missed, the marked recount method produces large overestimates of the population size, particularly if the probability of missing a nest on a subsequent survey is independent of the probability of missing it in the first survey. In other words, this is particularly true if a nest's visibility on a particular day depends more on the individual observer, the specific day or the specific decay stage the nest is at as opposed to invariant properties of the nest that would affect its visibility (e.g., nest height, surrounding vegetation.

The marked recount method performed better than the matrix method in detecting a population decline. In particular, provided our sampling recommendations are followed, the marked recount should be fairly sensitive in detecting large declines in population size, though smaller declines may still be difficult to easily detect. In order to prevent overreaction due to false positives obtained using this method (since in a non-declining population the method would find a decline 50% of the time and an increase 50% of the time), we suggest relying on a consistent pattern of decline over several months to confidently claim that a given population is declining. Economic analyses that explicitly incorporated the sensitivity of alternative sampling schemes to detect population declines, each scheme's cost, the cost of reacting to a false population decline and the cost of not reacting to a true population decline might provide more concrete guidance on whether nest count methods are an economically viable way to monitor orangutan populations, and could provide guidance on how those monitoring programs should proceed.

The results presented here should be interpreted cautiously as they depend on the specific parameters used. In particular, we only modeled some sources of variation in nest decay rates and did not simulate non-random orangutan movement. However, to the extent possible, the model's parameters were chosen carefully to mimic actual orangutan populations and their nests. Tests using a realistic range of values for these parameters produced qualitatively similar results.

Using nest surveys to estimate great ape population sizes is an inexact science. These results suggest that, for orangutans at least, effort and resources would be best spent on sampling approximately 0.26% of the study area and that additional resources would be better spent surveying more populations rather than increasing surveying effort within a particular population. These results could also inform studies aimed at other great ape species, though differences in the species' socioecological parameters suggest that extrapolation from our results should be done with caution. Additionally, these results suggest that at best one can expect an unbiased estimate of the population size within about 33% of the true population size about 50% of the time. To achieve better results for a single population, the results of many surveys over time could be averaged. However, these results suggest that nest survey methods will never provide quick, very precise population estimates and should be used cautiously to monitor populations, preferably in conjunction with other methods of detecting population change or detecting change in the threats facing the population.
